# The role of economic, educational and social resources in supporting the use of digital health technologies by people with T2D: a qualitative study

**DOI:** 10.1186/s12889-021-10325-7

**Published:** 2021-02-05

**Authors:** Sophie Turnbull, Patricia J. Lucas, Alastair D. Hay, Christie Cabral

**Affiliations:** 1 Centre for Academic Primary Care, Population Health Sciences, Bristol Medical School, University of Bristol, Canynge Hall, 39 Whatley Road, Bristol, BS8 2PS UK; 2 School for Policy Studies, University of Bristol, 8 Priory Road, Bristol, BS8 1TZ UK

**Keywords:** Health inequalities, Health equity, Digital divide, Digital health, Ehealth, Web interventions, Diabetes, Chronic conditions

## Abstract

**Background:**

Type 2 Diabetes (T2D) is a common chronic disease, with socially patterned incidence and severity. Digital self-care interventions have the potential to reduce health disparities, by providing personalised low-cost reusable resources that can increase access to health interventions. However, if under-served groups are unable to access or use digital technologies, Digital Health Technologies (DHTs) might make no difference, or worse, exacerbate health inequity.

**Study aims:**

To gain insights into how and why people with T2D access and use DHTs and how experiences vary between individuals and social groups.

**Methods:**

A purposive sample of people with experience of using a DHT to help them self-care for T2D were recruited through diabetes and community groups. Semi-structured interviews were conducted in person and over the phone. Data were analysed thematically.

**Results:**

A diverse sample of 21 participants were interviewed. Health care practitioners were not viewed as a good source of information about DHTs that could support T2D. Instead participants relied on their digital skills and social networks to learn about what DHTs are available and helpful. The main barriers to accessing and using DHT described by the participants were availability of DHTs from the NHS, cost and technical proficiency. However, some participants described how they were able to draw on social resources such as their social networks and social status to overcome these barriers. Participants were motivated to use DHTs because they provided self-care support, a feeling of control over T2D, and personalised advice or feedback. The selection of technology was also guided by participants’ preferences and what they valued in relation to DHTs and self-care support, and these in turn were influenced by age and gender.

**Conclusion:**

This research indicates that low levels of digital skills and high cost of digital health interventions can create barriers to the access and use of DHTs to support the self-care of T2D. However, social networks and social status can be leveraged to overcome some of these challenges. If digital interventions are to decrease rather than exacerbate health inequalities, these barriers and facilitators to access and use must be considered when DHTs are developed and implemented.

**Supplementary Information:**

The online version contains supplementary material available at 10.1186/s12889-021-10325-7.

## Background

Type 2 Diabetes (T2D) is a common chronic disease that creates a considerable burden to patients and health services [1–6]. A diagnosis of T2D results in widespread changes in the lives of the person with the condition as well as their families [1]. By their nature, chronic conditions cause illness over long periods and their management is complex and costly [3]. There is a social gradient to chronic illness, whereby people with lower Socio-Economic Status (SES) experience both a higher incidence and greater severity of chronic disease than those with higher SES [3, 7]. It has been proposed that this gradient is created by unequal access to resources, such as: knowledge, power, advantageous social connections, money, status and good quality healthcare [8–11]. People in more privileged social positions have greater access to these key resources that they can leverage to avoid risks to health and minimise the consequence of illness once it occurs [8–11]. Those in less privileged positions have fewer resources, which means they are less likely to have good control over their health and that there are greater barriers to managing illness [8–10, 12].

Digital Health Technologies (DHTs) are a resource that people with chronic conditions, like T2D, can use to help them to manage their condition. DHTs have the potential to reduce health disparities, by increasing access to personalised, low-cost health interventions, whilst reducing demand on an overstretched healthcare system [13–15]. The digital divide in terms of unequal internet access has narrowed across socio-economic and cultural groups, largely due to increased Smartphone ownership and the reduction in the cost of technology [16–19]. There is some evidence that DHTs can be acceptable, feasible [20–22], and effective [23] in populations that are traditionally viewed as underserved by health services. These interventions may also redress power imbalances between patients and Health Care Professionals (HCPs), by providing access to health information that was previously only available to clinicians [24].

However, there is some evidence that people from lower SES groups with fewer resources are less likely to access and use DHTs [25, 26]. Web-based health information has been found to be variable in quality, challenging to navigate and has mostly been developed to be used for people with high-school or greater reading ability [27, 28]. A qualitative study based in Australia found that people from lower socio-economic groups with less economic, cultural and social capital faced greater challenges accessing and using digital technology (not health related), which reinforced or increased existing disadvantage [29]. Participants with lower SES could not afford to purchase new technology (economic capital), found technology challenging use because of lower levels of education (cultural capital), and they did not have the social connections (social capital) to support the use of the technology [29]. Baum et al.’s (2014) study [29] provides a more sophisticated approach to the digital divide and social inequalities than considered in much intervention literature, but did not explore the impact of DHTs on the experience of living with and self-managing chronic conditions, specifically T2D.

This study was designed to explore: how and why people with T2D access and use DHTs to help them manage their condition and how experiences vary between individuals and social groups.

## Methods

The methodological orientation underpinning the study was an inductive approach drawing on aspects of grounded theory [30, 31]. Grounded theory involves the generation of theory and hypotheses, which are ‘grounded’ in data that has been collected and analysed systematically [30]. Ethical approval was granted from University of Bristol Faculty of Health Sciences Research Ethics Committee 27th April 2017. This research was conducted as a component of STs PhD, the full study details are available elsewhere [32].

### Participants

Participants were recruited face-to-face and by email from diabetes and community groups, focussing on groups that served Black, Asian and Minority Ethnic and lower income neighbourhoods. Adverts were placed in Diabetes UK online and print magazine, and ST spoke about the study on a Bristol radio station that focusses on serving ethnic minority groups. Adult participants were sought who had a diagnosis of T2D, had used DHTs to help them self-care for their condition and spoke and understood English. The recruitment materials were designed to seek out a range of experiences of DHTs including those who did not like them. Participants were told we were looking for people who had ever tried web-based tools or apps to help them manage their diabetes. The participant information sheet gave the following examples “web-based/internet sites or apps that helps you manage your condition and includes Diabetes websites (eg Diabetes UK), internet forums or apps you have downloaded onto your smart phone or tablet.” Our recruitment materials also described wearables such as Fitbits to reflect our interest in web-based tools such as monitors and wearables with accompanying apps or websites, in addition to stand-alone websites and apps. The recruitment materials also requested individuals interested in the study to complete a questionnaire (available in the Additional file 1) to enable purposive sampling of participants, allowing for the capture of a range of experiences across different social groups. Twenty-seven potential participants completed the questionnaire and interviews continued until data saturation was reach for the major themes, with twenty-one interviews completed.

### Procedure

Participants were provided with written information about the study before agreeing to interviews. At the beginning of each interview participants were given the opportunity to ask questions, were reassured that they were not obliged to take part and could choose not to answer any questions they felt uncomfortable about. Participants were asked to provide written consent before the interview began.

Semi-structured interviews were conducted by telephone, in participants’ homes and in a diabetes unit in a hospital. The interviews were conducted by one researcher (ST) and ranged from 35 min to 2 hours 13 min and were transcribed verbatim.

The development of the topic guide was informed by relevant theoretical frameworks and evidence from studies that have explored the experiences of living with a chronic illness, self-care and health inequalities. This included: chronic illness as an assault on personal identities; stigma in chronic illness; self-determination, control and the moral component of self-care; and the influence of the socio-economic context on how people adapt to a chronic illness diagnosis and their ability to self-care for their condition. There were three iterations of the topic guide, with minor changes around challenges of conducting self-care activities in the context of social gatherings (version 1.0 and the final version 1.3 available in Additional file 1). Field notes were taken during and after interviews.

### Analysis

Analysis was ongoing and iterative and began soon after data collection had started. Insights from analysis informed subsequent data collection and the topic guide was revised to reflect emerging themes from the analysis. Interviewing continued until data saturation was reached and no new data was arising in relations to the key themes. The interviews were recorded on encrypted audio-recorders and transferred to the University of Bristol secure servers where they were kept in accordance with the Data Protection Act 2018. Transcripts were anonymised, checked for accuracy and imported into NVivo for analysis. The data were analysed using the Thematic approach [33]. Some major themes were derived from theory prior to coding and further themes were derived from the data as they emerged. Three transcripts were coded by ST and were independently coded by two other authors (CC and PL). The lists of codes were reviewed in a meeting and ST, CC and PL reached a consensus on the list of themes. New themes emerging in subsequent transcripts were discussed in regular meetings with the team and the coding structure was further refined (coding tree available in Additional file 1). Participants were provided with a summary of the findings.

### Research team and reflexivity

#### Personal characteristics

This study was conducted as part of ST’s PhD, during which she received formal and informal training in qualitative methods and was supervised by senior academics with specialism in qualitative research (CC and PL). ST’s previous qualifications were a BSc in psychology and an MSc in neuropsychology and most of her training and experience is in quantitative methods, which may have had a bearing on the conduct and the interpretation of the interviews.

#### Relationship with participants

There was no prior relationship with the study participants before the study commenced. Participants interviewed in person would be aware that the interviewer (ST) was a white woman, in her thirties, who is relatively affluent, with no visible disabilities and a healthy weight. All would have known that the author was a student researcher at the University of Bristol. The participants knew that the study was about the use of technology to support the self-management of T2D but did not know the author was exploring differences by socio-economic and cultural groups.

The position taken by the ST was that DHTs have the potential to be beneficial for people with chronic conditions and there are likely to be socio-cultural differences in the way people access and use technology.

## Results

### Sample description

Twenty-one people were interviewed. One person expressed an interest in the study but chose not to proceed because they did not feel comfortable with the University standard procedure of data storage. The sample was diverse in terms of age (median 60 years, range 29–74), gender (11 men), socio-economic situation and household income. Two thirds (62%) had a University degree or equivalent and 17 (81%) participants identified as White British. The sample overview is in Table 1 and the individual participant profile in in Table 2.

**Table 1:** Participant characteristics

Participant characteristics	Male (*n*=11)	Female (*n*=10)
**Education, n (%)**		
Secondary school or equivalent (low education)	1 (9)	0 (0)
Intermediate between secondary level and university (eg, NVQ3–5^a^, diploma, and apprenticeship (low education)	5 (46)	2 (20)
University degree or equivalent (High education)	5 (46)	8 (80)
**Estimated household income in the last year (before tax and not including benefits), £, n (%)**		
Lowest income < 16,000 and/or eligible for means tested benefits	3 (27)	0 (0)
Low income 16,000–24,999	1 (9)	4 (40)
Mid income 25,000–34,999 (33,331-46,662)	3 (27)	0 (0)
High income 35,000–44,999	0 (0)	2 (20)
Highest income > 45,000	2 (18)	2 (20)
Prefer not to say	2 (18)	2 (20)
**Use of digital interventions, n (%)**		
Lighter (≤2 interventions)	7 (64)	5 (50)
Heavier (> 2 interventions)	4 (36)	5 (50)
**Home neighborhood deprivation**^**b**^**, n (%)**		
1 Most deprived	1 (9)	2 (20)
2 Lower SES^c^	2 (18)	1 (10)
3 Mid SES	3 (27)	1 (10)
4 Higher SES	1 (9)	2 (20)
5 Highest SES	4 (36)	3 (30)
Not available	0 (0)	1 (10)
**Age, years, n (%)**		
21–40	1 (9)	1 (10)
41–60	4 (36)	5 (50)
61–70	6 (55)	4 (40)
71–80	2 (18)	1 (10)

^a^NVQ3–5: National Vocational Qualification levels 3 to 5

^b^Indices of multiple deprivation score derived from the participant’s home post code were used to determine the participant’s neighbourhood deprivation within the United Kingdom, and the quintile is given

^c^SES: socioeconomic status

**Table 2:** Profile of individual participants

ID	Gender	Age range	Ethnicity	Highest level of education	Estimated household income last year (before tax and not including benefits)	IMD quintile
10	Female	61–70	White- British	Intermediate between secondary level and university (e.g. NVQ3–5, diploma, apprenticeship)	£16,000 to £24,999	1
11	Female	41–60	White- British	University degree or equivalent	£35,000 to £44,999	5
20	Male	71–80	White- British	Intermediate between secondary level and university (e.g. NVQ3–5, diploma, apprenticeship)	£25,000 to £34,999	2
22	Male	61–70	White- British	Secondary school or equivalent	£25,000 to £34,999	5
23	Male	21–40	Asian or Asian British-Indian	University degree or equivalent	<£16,000 and/or eligible for means tested benefits	3
24	Female	21–40	Asian or Asian British-Indian	University degree or equivalent	£16,000 to £24,999	2
26	Male	41–60	Asian or Asian British-Indian	Intermediate between secondary level and university (e.g. NVQ3–5, diploma, apprenticeship)	£16,000 to £24,999	2
27	Male	41–60	White- British	Intermediate between secondary level and university (e.g. NVQ3–5, diploma, apprenticeship)	Prefer not to say	5
28	Male	61–70	White- British	Intermediate between secondary level and university (e.g. NVQ3–5, diploma, apprenticeship)	>£45,000	3
29	Male	61–70	White- British	University degree or equivalent	>£45,000	5
30	Female	41–60	White- British	University degree or equivalent	>£45,000	NA
31	Female	41–60	White- British	Intermediate between secondary level and university (e.g. NVQ3–5, diploma, apprenticeship)	Prefer not to say	5
33	Female	41–60	White- British	University degree or equivalent	Prefer not to say	5
34	Male	41–60	White- British	Intermediate between secondary level and university (e.g. NVQ3–5, diploma, apprenticeship)	£25,000 to £34,999	1
35	Female	61–70	Other-White European, with mixed racial ancestry	University degree or equivalent	£35,000 to £44,999	3
36	Male	41–60	White- British	University degree or equivalent	<£16,000 and/or eligible for means tested benefits	4
37	Female	61–70	White- British	University degree or equivalent	£16,000 to £24,999	4
38	Male	61–70	White- British	University degree or equivalent	<£16,000 and/or eligible for means tested benefits	5
40	Female	71–80	White- British	University degree or equivalent	£16,000 to £24,999	4
41	Female	41–60	White- British	University degree or equivalent	>£45,000	1
42	Male	71–80	White- British	University degree or equivalent	Prefer not to say	3

The sample was self-selecting for those who had tried using digital technology to support the management of their condition. However, not all people in the sample were technophiles (people who like technology). Participants ranged from those who used one intervention to those who used multiple digital interventions (up to 7), and in one case the participant had tried digital interventions but had stopped using them because she did not find them helpful (ID 24). The median number of DHTs used by the participants was two. We classified participants who had used two or fewer interventions as lighter users and those who used more than two interventions as heavier users. Twelve participants (57%) were lighter users (≤2 intervention) and nine (43%) were heavier users (> 2 interventions) of DHTs (Table 1).

### Digital health technologies used

The DHTs identified by participants included web-based tools, and stand-alone apps and websites. This included: Blood Glucose Monitors (BGMs) with online components (e.g. an app or website to view the data), wearable technology (e.g. Fitbits), online access to electronic health records, diabetic specific and general health websites and apps. Most people did not use interventions designed specifically for people with diabetes, but rather used DHTs designed to support healthy living and social connectivity. Wearable fitness trackers were the most commonly used intervention (16 participants) and apps that tracked nutrition or fitness (11 participants). The diabetes specific interventions were the BGMs (Dario meter, Freestyle Libre, Trueyou mini) used by ten participants (five supplied by HCPs and five purchased privately), and three different apps each used by one participant (Diabetes diary, IBG star app and Habits- South Asian specific diabetes app).

The way in which the participants used the range of DHTs fell into two broad groups. The first was to learn more about diabetes and to find guidance on how to manage it. Participants predominantly used websites (including forums and websites such as Diabetes UK) and apps to achieve this goal. The second was to help them understand more about their bodies, and to feel more in control. To do this they: used feedback from blood glucose monitors and wearables, accessed their electronic health records to track how their blood glucose test results changed over time, and used apps and websites to track data on diet and exercise. The participants described changing their behaviours in response to this feedback, for example, by changing their diet or increasing their level of exercise.

### How people learned about digital interventions

Few participants described learning about DHTs from HCPs, and most felt that HCPs had limited knowledge of technology that could support their diabetes self-care. They talked about educating HCPs who “*don’t get the technology*” (ID 10, white female, Low Ed) about what is available to the public and their benefits. Only one person mentioned that the Food Smart app had been recommended to them the “*first [NHS] health visit that [he] had from this wellbeing thing*”(ID 22, white male, Low Ed).

Instead, participants described learning about DHTs that might support their self-management through searching the internet, social networks, support groups and online communities and forums. Participants talked about how they ‘googled’ DHTs, navigated apps stores and products and sought out expert advice. Many participants initially found out about technology through friends and family. Participants took advice on DHTs from those whose opinions they trusted and valued, because they were friends, were perceived to have higher status, or because they appeared to have professional knowledge. One man described how he learnt about the Change for Life app through “*very knowledgeable”* people in the diabetes research focus group he attends:

*… they have a much more in-depth, er, understanding of things. And they present more problems, and ask more questions, and say things that we wouldn’t dream of saying. (ID 28, White male, Low Ed)*

Group membership influenced the type of technology people heard about. Participants who were involved in community-based diabetes support groups and diabetes research groups described finding out about technology themselves from magazine articles, talks and conferences and hearing about them from other group members. They also had ‘professionals’ representing digital health companies like Abbott attend their meetings. Online communities and forums fulfilled a similar purpose to physical support groups in spreading information about innovations in technology.

*I’m sort of active in the diabetes online world ( … ) there are always people there talking about new innovations. (ID 33, white female, High Ed)*

### How people acquire technology

#### In context with health services

Many participants believed that limited resources in the NHS prevented them from accessing DHTs to support their diabetes self-care. This came across particularly strongly in the context of BGMs. Some participants described being provided BGMs while others described how the NHS “*refused to give [them] a meter*” (ID 27, white male, Low Ed). Those who were not supplied monitors felt that the NHS was limiting availability of BGMs to people with T2D because of budgetary restraints or perceived need.

*it’s disgraceful really that these technologies, the quite basic technologies, are so blinking expensive that people feel they have to be cut. You know, things that help people self-manage. Because as soon ( … ) you get better educated and self-managed things improve, but, you know, we live in a time when that doesn’t count really. (ID 37, white female, High Ed)*

Some participants privately bought BGMs because they were not supplied by their HCP or because they felt that the equipment provided was not adequate for their needs.

*I belong to a forum called, Diabetes.co.uk. Erm, and, erm, I learnt most of what I know about diabetes on there. Erm, and, there were people talking about how to fund your own blood glucose testing by using cheap meters and whatever. And pay for them privately rather than have a prescription. And I’d done that. (ID 41, white, female, High Ed)*

Participants described having negative reactions from HCPs about their use of BGMs when they had bought one for themselves, rather than being supplied or prescribed one on the NHS. One woman talked about being frustrated with the response from her doctor about Freestyle Libre, who was critical because *“it doesn’t meet with any approval in this neck of the woods.”* (ID 41, white female, High Ed). One participant described not being provided with a BGM because the nurse felt having access to a BGM may mean he ended up “*in an even deeper hole”* with his health-related anxiety (ID 27, white male, Low Ed). However, other technology (such as digital dietary and activity aids) used to support self-care behaviours appeared to elicit more positive reactions: *“I showed them [Diabetes Diary app] to a doctor ( …*) *he thought it was an excellent idea”* (ID 20, white male, Low Ed).

#### Barriers and facilitators to access

The main reported barriers to privately accessing DHT were cost and technical proficiency. However, some participants described how they were able to draw on social resources to overcome these barriers.

The cost of DHT was prohibitive for some participants. Participants described how they had considered buying expensive technology like the Freestyle Libre, but the high cost meant it was “*a no-go*” (ID 40, white female, low income). One woman talked about how Fitbits had become less affordable “*This one was £60, that’s the cheapest. Now they’ve gone up to about £90 I think*” (ID 10, white female, low income). Some used expensive technology (such as the Freestyle Libre) but limited its use to minimise expense, only using it “*when things were going to be changing*” (ID 42, white male). Others described using DHT that were free to download onto their smartphones.

Participants described how access to DHTs was facilitated by people in their personal networks. They talked about having access to technology such as smartphones and watches through being given “*a very generous gift*” (ID 41, white female, highest income) and through perks from work such as company phones that are free to use. One participant described how her personal trainer got her to use an app (MyFitnessPal) to keep a track of what she was eating to “*really understand the diabetes more*” (ID 37, white female, High Ed, low income).

Group membership provided benefits which included access to DHTs. Those who were members of diabetes support groups talked about receiving discounts off expensive DHTs and being offered free samples.

*people within the group have availed themselves of it [Freestyle Libre], because we did get some, erm, free vouchers from the rep, and these were distributed within the group. (ID 42, white male, High Ed)*

Some people self-identified as early adopters and technophiles, while other people felt less able to navigate new innovations but were still using DHTs. There was a suggestion that limitations in the individual’s knowledge of and skill to use technology could be overcome by support from people in their social network; where people with technology knowledge and skills could act as tech buddies to help the participants overcome issues with usability.

*now I couldn’t load it, and luckily I’ve got a daughter and a wife who is sort of techie, you know. I’m a bit of a technophobe … (ID 10, White Female, Low Ed)*

*What we say to our support group members is, those who are not so smart, for phone, kind of geeks, just go and tell you family members to help you. (ID 26, Asian British-Indian Male, Low Ed)*

Social capital seemed to help some participants be able to gain better access to technology. A man talked about how his work as “*a Microsoft partner”* meant he was able to negotiate getting replacement technology when his failed because he felt confident with technology companies (ID 36, Male, White, High Ed, lowest income). Another man used his role as lead of a South Asian diabetes support group to gain pre-launch access to a culturally sensitive app for himself: “*it hadn’t reached the iPhone yet. ( …) I said to the company [making the app], “Well let’s, erm, you’re going to launch it, let’s pilot it within our groups, to see … The effectiveness, to see how, what people think.”* (ID 26, Asian British-Indian male, Low Ed and income).

### Why people select and use technology

Participants were motivated to use DHT because they provided information about how to manage diabetes, self-care support, a feeling of control over T2D, and personalised advice or feedback. The selection of technology was also guided by participants’ preferences and what they valued in relation to technology and self-care support, and these in turn were influenced by age and gender.

Participants described how DHTs gave them a sense of being in control of their condition by providing self-care support and feedback. They talked about how BGMs kept them on “*the straight and narrow*” with the diet recommended to people with T2D by the NHS [34], by providing personalised feedback, meaning “*you have nowhere to hide from that evidence*” of how food impacts blood glucose levels. (ID 33, white female, High Ed). Others talked about how the feedback from wearables like Fitbits had “driven” them to increase their activity levels and “change my lifestyle as a result of trying to get that 7000 steps.” (ID 27, white male, Low Ed) Access to BGMs was particularly important to the participants because many felt that this technology gave them greater control over their blood glucose levels or diabetes in general.

*without those two things [blood glucose meter and Freestyle Libre], I wouldn’t be in control of my blood glucose. ( …) my HbA1c, would be up in the, in the, erm, diabetic range. There’s no way I could keep this level of control (ID 41, white female, High Ed, highest income)*

DHTs were valued by many participants because they felt that the personalised information provided was more beneficial than “*one fits all” (ID 27)* guidelines issued by HCPs and in structured education courses. Participants talked about turning to DHTs and forums because they offered tailoring to different culturally specific needs, personal diet preferences and learning styles that were not catered for in community-based education courses and leaflets from HCPs they had experienced.

“*I thought, “Wow this is something I’ve been looking for, for a while” [Habits South Asian specific diabetes app] And it’s now here, so we have to take advantage (ID 26, Asian British-Indian male, Low Ed)*

*when I was diagnosed diabetic, I wasn’t offered a course and I didn’t push for it. Because I have, I had heard feedback from other people on the forum who had gone on said course … and found it absolutely useless, because it just pushed carbs. (ID 41, white female, High Ed)*

*how appropriate that style or level of learning is for any of those people [in the DESMOND course], never mind all of them, it’s gonna be suboptimal because ( …) people aren’t gonna get the same things out of it. Some will get m-much more than others … (ID 33, white female, High Ed)*

Some participants felt that physical courses and DHTs were complementary, fulfilling different roles in relation to education and practical support.

*whatever you get from a sort of structured education programme ( …) I think those can only be the principles and brushstrokes(...) what you get from the self-stuff is, like, colouring it in, getting, getting the detail. (ID 33, white female, High Ed)*

There was a perception from some participants that technology could not replace current effective non-digital interventions. A few of the interviewees talked about the benefits of a physical courses over DHTs, including having someone to “show people how” to do an activity (ID 26, Asian British-Indian male). One man felt the physical prompts he used for taking medication in pill form (e.g. medicine dosset boxes) could not be easily replaced by technology, but found apps helpful for tracking his intake of insulin where no physical prompts were available.

*it’s [apps] of no benefit if you are just taking medicine, because it doesn’t record … the way you take your metformin because the dosset box you can see when they have popped ( …) So, so taking insulin was a driver to get an app that would thoroughly keep a record of when I had two, or done something … (ID 20, white male, 71–80 yrs age range)*

There were mixed preferences with regard to digital social forums for social support. Several participants described receiving all the support they needed from online forums.

*I’ve made quite a few friends on there [Diabetes.co.uk], erm, and we, we interact separately from the forum. (ID 41, white female, 41–60 yrs age range, High Ed)*

Other participants talked about how people would miss out emotional support and learning from other people with diabetes, which they felt “*an app doesn’t replicate*” (ID 29, white male, 61–70 yrs. age range). A few were very negative about sharing their experiences and seeking support on digital social forums.

*the idea of sort of going onto, er, onto a sort of social website, to say that, you know, “I’m feeling great today, or not sort of great today”. And then waiting for somebody else, to comment on it, that, that’s, that seems just pretty futile, and narcissistic. (ID 29, white male, 61–70 yrs age range, High Ed)*

Technology was described by many of the (older) participants as something that young people use and older people resist: “*I don’t think it’s any point trying to tell an 85-year-old about Fitbits*. *But* *someone who’s sort of, has an understanding, try it, see if it works for you*.” (ID 27, white male, 41–60 yrs. age range). For some participants, differences in use of technology between older and younger people had been observed as well as perceived. There was the view that younger people had a better understanding of technology and some of the DHTs were better suited to the way younger people interact with technology such as “*chat, er, forums and things like that*” (ID 37, white female, 61–70 yrs. age range).

In contrast the two younger participants in the sample (21–40 yrs. age range) talked about the benefits of physical interventions over DHTs. The younger man felt that non-digital interventions increased his opportunities to make social connections and used the discussion of health apps as a conversation starter with people at the gym:

*I would like to do a course [Man vs Fat], and that would sort of encourage me to meet other people, but also, to ( …) share ideas, on what works for them, and what’s been quite useful (ID 23, Asian British-Indian male, 21–40 yrs age range, High Ed, lowest income)*

The younger woman felt apps were not very good compared to in-person courses like LEAP and Weightwatchers “*Cause the whole point is you got to be physical*” (ID 24). She was the only participant that felt none of the DHTs she had tried had been helpful for the management of diabetes. She characterised DHTs as being for people who were already “*independent in their own exercise*” (ID 24). She did express the feeling that she had different requirements than others on the diabetes support course because they were much older, but she found the quick progress she made relative to the older attendees motivating:

*I found it [LEAP] really good but I was like the youngest one there. So everyone else was like, quite sedentary. And I found it really easy to lose weight and, erm, and they all just like, hmm. (ID 24, Asian British-Indian female, 21–40 yrs age range, High Ed, low income)*

Male and female participants emphasised different concerns about technology. Many of the men in the group had concerns about data security and with what companies were doing with their personal details or whether “*nasty people*” (ID 36) could hack and used their data maliciously. Male participants also talked about some technology feeling insidious, “*like you are being watched*” (ID 22, white male, Low Ed). Some of the female participants spoke about challenges with establishing which online sites were credible sources of information but did not bring up issues about security.

## Discussion

### Summary of main findings

Participants described how they: learnt about, acquired, and used technology to support the self-management of their T2D. Participants rarely learned about DHTs from HCPs and did not perceive HCPs as knowledgeable about self-care technology. Instead they sought information from their personal social networks and diabetes support groups (in person and online). The main barriers to accessing and using DHT described by the participants were availability of DHTs from the NHS, cost and technical proficiency. However social resources such as social networks and social capital could be leveraged to overcome some of these barriers. Participants gained access to technology through their personal networks through gifts and work perks. Group membership provided benefits which included access to discounts off expensive DHTs and being offered free samples. Social capital was used to negotiate getting replacement technology and to gain pre-launch access to apps. Participants described how a lack of digital skills could be barrier to the use of DHT, but could be overcome by drawing on support from tech buddies in their social network. Participants used DHTs because they provided: information about how to manage diabetes, self-care support, a feeling of control over T2D, and personalised advice or feedback. They selected DHT because they provided personalised information that could be tailored to culturally specific needs, diet preferences and learning styles, and could provide social support. Some participants felt that non-DHTs were better at providing some aspects of support for T2D management, such as ‘how to’ training and emotional support. Although technology was constructed by many participants as something that young people use and older people resisted, the younger participants in the sample valued the face-to-face support and networking available from non-digital interventions. Men and women expressed different concerns about technology: most of the men brought up worries about data security, which was never mentioned by women.

### In the context of other literature

This study has shown how economic, educational (digital skills) and social resources (social networks and social capital) can influence the access and use of technology for self-care by people with T2D. This agrees with previous evidence that has indicated that people with fewer resources are less likely to access and use digital technologies [25, 26, 29]. As with this study, Baum et al. (2014) found that those with fewer economic, educational and social resources encountered more challenges accessing and using digital technology (not health related) and that social networks facilitated access [29]. They also found evidence of digital exclusion being amplified by social exclusion [29], which was not reported by participants in this study.

The finding in this study that difference in resources influenced how people learnt about and accessed health technology, supports theories of health inequalities including the theory of fundamental causes and social capital theory. The theory of fundamental causes suggests that there is a social gradient in the control people have over their lives that it is mediated by disparities in the array of resources available to them [8–10]. The resources include: power, advantageous social connections, money, knowledge and prestige [8–10]. This study provided examples of how each of these resources were drawn on to overcome barriers to accessing DHTs that were used by participants to gain a feeling of control over their diabetes. DHTs can also be used to support social status through projection of a positive identity and avoidance of the stigmatised illness identity [35]. Social capital theory addresses inequities at a community level, and proposes there is a social hierarchy in ‘the ability of actors to secure benefits by virtue of membership in social networks and other social structures’ [36]. This theory suggests that belonging to a social network, provides access to resources and benefits that individuals would not have on their own [37]. This study highlighted the role of membership to social groups (e.g. diabetes groups, research groups and online forums) in providing knowledge about technology and shortcuts to accessing new and helpful innovations. This demonstrates that traditional measures of deprivation such as education, occupation and household income, are not sufficient to encapsulate the resources people had available to them.

There was also some evidence of ‘bridging social capital’ through memberships to these groups. Bridging is the connections that link people across different networks or social groupings (such as ethnicity, occupational class, or religion), which are responsible for the transmission of information and resources [37–40]. Bridging occurred through diabetes support groups, involvement in research groups and online forums. A clear example of this is where a man from a traditional occupational working-class background with lower education learned about technology he had ‘never heard of’ through others who were ‘very knowledgeable’ in his research group. He may not have had the opportunity to learn about these innovations through his own personal network and gained access to the knowledge of people from different occupational and educational backgrounds.

### Strengths and limitations

To the authors knowledge, this is the first study to explore how people with T2D choose technology to support to the self-care and their experiences using DHTs. Participants were offered interviews in person, by video call and telephone. The majority selected telephone interviews. Telephone interviews may have been more appealing to participants because of a perception of a higher degree of anonymity [41, 42], while discussing sensitive issues and in the context of their diagnosis of a stigmatised condition [43, 44]. Complete audio data was recorded for all interviews except one telephone interview where the first 10 min were lost because the recording device malfunctioned. There were no further issues with lost data. In three phone interviews family members (children and partners) were around the person being interviewed, which may have affected the content of the interview. The participants were not asked to comment on the transcripts. Double coding of a subset of interviews by two members of the team and ongoing discussion about coding structure ensured the coding scheme was robust. Multiple views of the data promote confidence in the credibility of the findings [45]. A diverse range of experiences and opposing sides of arguments were identified and presented.

Some caution should be exercised in the transferability of the findings to other settings or populations. Despite targeted efforts made to recruit a diverse sample in terms of ethnicity and religion most of the participants identified as White-British and Christian. Consequently, thematic analysis may not capture the range of experiences of those from minority ethnic groups. The decision to restrict interviews to English were due to a lack of resources for interpreting, and in response to challenges with conducting cross-language qualitative research [46]. However, this may have created a barrier to study entry for some groups. People who expressed an interest in the study were mostly adults > 51 years who had taken an interest in technology and were engaged in the innovations. However, the participants were not all technophiles. Those who had previously used technology but were no longer using technology were also actively sought and were present in the group, as were lighter users of technology. Those who had never used technology were not included because the main aim of the study was to understand differences in experiences of using DHTs by people from different socio-cultural backgrounds. This is likely to have excluded some groups of people who have historically been found to have lower access to the internet including; older people, those from minority ethnic groups, with lower SES and those living in remote geographical regions [47, 48].

In this study we chose to classify participants as heavy or light users of DHTs depending on the number of DHTs they had engaged with. However, we acknowledge that use is difficult to quantify. We considered asking the participants about their level of daily engagement with the DHTs they used, but felt their judgement would reflect their confidence and knowledge as much as the extent of their use.

### Implications for future research, policy and clinical practice

This research has highlighted the limitations of using individual measures of inequalities (such as education and income) to encapsulate the social determinants of health and resources available to a person. These measures did not account for the importance of membership to social groups (e.g. diabetes groups, research groups and online forums) and how these supported access to knowledge about technology, provided shortcuts to accessing new and helpful innovations, and support to overcome issues with usability (tech buddies) [37–40, 49]. Research into health inequalities should consider the important role of social and community assets in the access and use of health interventions.

The training and availability of tech buddies may reduce barriers to accessing health technology caused by a lack of knowledge about available digital interventions, and how to access and use them [50]. As NHS policy begins to encourage greater adoption of digital interventions, primary care HCPs with oversight of those with chronic conditions are likely to play a role in supporting people to access and use these interventions. However, in the context of growing financial and workforce pressures [50, 51] it may be useful for HCPs to be able to signpost patients to trained ‘tech buddies’ in community services. People diagnosed with chronic conditions could be linked with trained ‘tech buddies’ who can discuss potential technological support with them, and troubleshoot issues with technology. Currently available peer support schemes, and social prescribing programmes have been found to be acceptable and beneficial for people with chronic conditions [52, 53].

## Conclusions

This research indicates that low levels of digital skills and high cost of some DHTs can create barriers to the access and use of DHTs to support the self-care of T2D. However, social networks and social status can be leveraged to overcome some of these challenges. If DHTs are to decrease rather than exacerbate health inequalities, these barriers and facilitators to access and use must be considered when interventions are developed and implemented. The training and availability of tech buddies may reduce barriers to accessing DHTs caused by a lack of knowledge about available interventions, and how to access and use them.

## Supplementary Information

**Additional file 1.** Qualitative study questionnaire.

## Data Availability

Anonymised interview transcripts are available from the corresponding author on reasonable request.

## Abbreviations

BGM: Blood Glucose Monitors
DESMOND: Diabetes Education and Self-Management for Ongoing and Newly Diagnosed
DHT: Digital Health Technology
HCP: Health Care Practitioners
NHS: National Health System
NICE: National Institute For Health And Care Excellence
LEAP: Let’s Empower and Prepare
SES: Socio-Economic Status
T2D: Type 2 Diabetes
UK: United Kingdom

## References

[1] Alonso J, Ferrer M, Gandek B, Ware JE, Aaronson NK, Mosconi P, Rasmussen NK, Bullinger M, Fukuhara S, Kaasa S (2004). Health-related quality of life associated with chronic conditions in eight countries: results from the International Quality of Life Assessment (IQOLA) Project. Qual Life Res.

[2] WHO (2004). The global burden of disease: 2004 update. WHO library cataloguing-in-publication data.

[3] Department of Health (2012). Long-term conditions compendium of information.

[4] Marmot M (2007). Achieving health equity: from root causes to fair outcomes. Lancet.

[5] Goodwin N, Curry N, Naylor C, Ross S, Duldig W (2010). Managing people with long-term conditions.

[6] Lewis R, Dixon J (2004). Rethinking management of chronic diseases. BMJ.

[7] Furler J, Harris M, Rogers A (2011). Equity and long-term condition self-management. Chronic Illn.

[8] Phelan JC, Link BG, Diez-Roux A, Kawachi I, Levin B (2004). “Fundamental causes” of social inequalities in mortality: a test of the theory. J Health Soc Behav.

[9] Phelan JC, Link BG, Tehranifar P (2010). Social conditions as fundamental causes of health inequalities: theory, evidence, and policy implications. J Health Soc Behav.

[10] Link BG, Phelan J. Social conditions as fundamental causes of disease. J Health Soc Behav. 1995;(Extra Issue):80–94. 7560851

[11] Marmot M (2004). Status syndrome: how your social standing affects our health and longevity.

[12] Ellis J, Boger E, Latter S, Kennedy A, Jones F, Foster C, Demain S (2017). Conceptualisation of the ‘good’ self-manager: a qualitative investigation of stakeholder views on the self-management of long-term health conditions. Soc Sci Med.

[13] Castle-Clarke S, Fund Ks (2018). What will new technology mean for the NHS and its patients: Four big technological trends. NHS at 70.

[14] Murray E, Burns J, See Tai S, Lai R, Nazareth I. Interactive Health Communication Applications for people with chronic disease. Cochrane Database Syst Rev. 2005;(4):CD004274. 10.1002/14651858.CD004274.pub4. 10.1002/14651858.CD004274.pub4 PMC13184810 16235356

[15] Muñoz RF (2010). Using evidence-based internet interventions to reduce health disparities worldwide. J Med Internet Res.

[16] Stellefson M, Chaney B, Barry AE, Chavarria E, Tennant B, Walsh-Childers K, Sriram PS, Zagora J (2013). Web 2.0 chronic disease self-management for older adults: a systematic review. J Med Internet Res.

[17] Stellefson M, Chaney B, Chaney D (2008). The digital divide in health education. Am J Health Educ.

[18] Poushter J, Center PR (2016). Smartphone ownership and internet usage continues to climb in emerging economies but advanced economies still have higher rates of technology use. Numbers, facts and trend shaping the world.

[19] Latulippe K, Hamel C, Giroux D (2017). Social health inequalities and ehealth: a literature review with qualitative synthesis of theoretical and empirical studies. J Med Internet Res.

[20] Pekmezi DW, Williams DM, Dunsiger S, Jennings EG, Lewis BA, Jakicic JM, Marcus BH (2010). Feasibility of using computer-tailored and internet-based interventions to promote physical activity in underserved populations. Telemed J E Health.

[21] Gustafson DH, Hawkins RP, Boberg EW, McTavish F, Owens B, Wise M, Berhe H, Pingree S (2002). CHESS: 10 years of research and development in consumer health informatics for broad populations, including the underserved. Int J Med Inform.

[22] Starren J, Hripcsak G, Sengupta S, Abbruscato CR, Knudson PE, Weinstock RS, Shea S (2002). Columbia University’s Informatics for Diabetes Education and Telemedicine (IDEATel) project: technical implementation. J Am Med Inform Assoc.

[23] Turnbull S, Cabral C, Hay A, Lucas PJ (2020). Health Equity in the Effectiveness of Web-Based Health Interventions for the Self-Care of People With Chronic Health Conditions: Systematic Review. J Med Internet Res..

[24] Murray E (2012). Web-based interventions for behavior change and self-management: potential, pitfalls, and progress. Med 2 0.

[25] Yu L (2006). Understanding information inequality: Making sense of the literature of the information and digital divides. J Librariansh Inf Sci..

[26] van Dijk J (2005). The deepening divide: inequality in the information society.

[27] Berland GK, Elliott MN, Morales LS, Algazy JI, Kravitz RL, Broder MS, Kanouse DE, Muñoz JA, Puyol J-A, Lara M (2001). Health information on the internet: accessibility, quality, and readability in English and Spanish. JAMA.

[28] Gilmour JA (2007). Reducing disparities in the access and use of Internet health information. A discussion paper. Int J Nurs Stud.

[29] Baum F, Newman L, Biedrzycki K (2014). Vicious cycles: digital technologies and determinants of health in Australia. Health Promot Int.

[30] Strauss A, Corbin JM (1997). Grounded theory in practice.

[31] Thomas DR (2003). A general inductive approach for qualitative data analysis.

[32] Turnbull S (2019). The influence of digital self-care interventions on health inequality in high burden chronic health conditions.

[33] Braun V, Clarke V (2006). Using thematic analysis in psychology. Qual Res Psychol.

[34] Food and keeping active-type 2 diabetes [https://www.nhs.uk/conditions/type-2-diabetes/food-and-keeping-active/].

[35] Turnbull S, Lucas P, Hay AD, Cabral C. The role of digital health interventions as a resource for people with type 2 diabetes to develop self-care expertise, adapt to identity changes and influence how they are viewed by others: a qualitative study. JMIR. 2020. 10.2196/21328 PMC7781797 33346733

[36] Portes A (1998). Social capital: its origins and applications in modern sociology. Annu Rev Sociol.

[37] Eriksson M. Social capital and health–implications for health promotion. Glob Health Action. 2011;4(1):5611. 10.3402/gha.v4i0.5611. 10.3402/gha.v4i0.5611 PMC3036711 21311607

[38] Putland C, Baum F, Ziersch A, Arthurson K, Pomagalska D (2013). Enabling pathways to health equity: developing a framework for implementing social capital in practice. BMC Public Health.

[39] Portes A (2000). The two meanings of social capital. Sociol Forum.

[40] What is bridging social capital? [https://www.socialcapitalresearch.com/what-is-bridging-social-capital/].

[41] Greenfield TK, Midanik LT, Rogers JD (2000). Effects of telephone versus face-to-face interview modes on reports of alcohol consumption. Addiction.

[42] Sturges JE, Hanrahan KJ (2004). Comparing telephone and face-to-face qualitative interviewing: a research note. Qual Res.

[43] Drabble L, Trocki KF, Salcedo B, Walker PC, Korcha RA (2016). Conducting qualitative interviews by telephone: Lessons learned from a study of alcohol use among sexual minority and heterosexual women. Qual Soc Work.

[44] Linnemayr S, Jennings Mayo-Wilson L, Saya U (2021). HIV Care Experiences During the COVID-19 Pandemic: Mixed-Methods Telephone Interviews with Clinic-Enrolled HIV-Infected Adults in Uganda. AIDS Behav..

[45] Sandelowski M (1995). Sample size in qualitative research. Res Nurs Health.

[46] Squires A (2009). Methodological challenges in cross-language qualitative research: a research review. Int J Nurs Stud.

[47] Hardiker NR, Grant MJ (2011). Factors that influence public engagement with eHealth: a literature review. Int J Med Inform.

[48] Gibbons MC (2005). A historical overview of health disparities and the potential of ehealth solutions. J Med Internet Res.

[49] Bourdieu P (1986). The forms of capital.

[50] Designing and assessing digital health services [https://www.nhsx.nhs.uk/key-tools-and-info/designing-and-building-products-and-services/].

[51] Boudreaux ED, Waring ME, Hayes RB, Sadasivam RS, Mullen S, Pagoto S (2014). Evaluating and selecting mobile health apps: strategies for healthcare providers and healthcare organizations. Transl Behav Med.

[52] Heisler M (2010). Different models to mobilize peer support to improve diabetes self-management and clinical outcomes: evidence, logistics, evaluation considerations and needs for future research. Fam Pract.

[53] Moffatt S, Steer M, Lawson S (2017). Link Worker social prescribing to improve health and well-being for people with long-term conditions: qualitative study of service user perceptions. BMJ Open..

