# Supplementary material for: The role of economic, educational and social resources in supporting the use of digital health technologies by people with T2D: a qualitative study
Source: BMC Public Health. 2021 Feb 5;21:293. doi: 10.1186/s12889-021-10325-7 (PMC7863320; doi:10.1186/s12889-021-10325-7)
Supplement: Supplementary file 1 — Additional file 1. Qualitative study questionnaire. [file 12889_2021_10325_MOESM1_ESM.docx]

Appendix

Qualitative study questionnaire

**Topic guide**

**Version 1.0 (original version)**

**Version 1.3 (final version)**
